# The interrelated effect of sleep and learning in dogs (*Canis familiaris*); an EEG and behavioural study

**DOI:** 10.1038/srep41873

**Published:** 2017-02-06

**Authors:** Anna Kis, Sára Szakadát, Márta Gácsi, Enikő Kovács, Péter Simor, Csenge Török, Ferenc Gombos, Róbert Bódizs, József Topál

**Affiliations:** 1 Institute of Cognitive Neuroscience and Psychology, Hungarian Academy of Sciences, Budapest, Hungary; 2 Institute of Behavioural Sciences, Semmelweis University, Budapest, Hungary; 3 MTA-ELTE Comparative Ethology Research Group, Budapest, Hungary; 4 Department of Ethology, Eötvös Loránd University, Budapest, Hungary; 5 Department of Ecology Faculty of Veterinary Sciences, Szent István University, Budapest, Hungary; 6 Department of Cognitive Science, Budapest University of Technology and Economics, Budapest, Hungary; 7 Institute of Psychology, Eötvös Loránd University, Budapest, Hungary; 8 Department of General Psychology, Pázmány Péter Catholic University, Budapest, Hungary.

## Abstract

The active role of sleep in memory consolidation is still debated, and due to a large between-species variation, the investigation of a wide range of different animal species (besides humans and laboratory rodents) is necessary. The present study applied a fully non-invasive methodology to study sleep and memory in domestic dogs, a species proven to be a good model of human awake behaviours. Polysomnography recordings performed following a command learning task provide evidence that learning has an effect on dogs’ sleep EEG spectrum. Furthermore, spectral features of the EEG were related to post-sleep performance improvement. Testing an additional group of dogs in the command learning task revealed that sleep or awake activity during the retention interval has both short- and long-term effects. This is the first evidence to show that dogs’ human-analogue social learning skills might be related to sleep-dependent memory consolidation.

Sleep is a fundamental, but compared to the awake processes often neglected, behavioural state present in almost all vertebrate species^1^. Despite the intertwined nature of sleep and awake states^2^, and the widely accepted notion that sleep has a vital function, there is still no general, unifying and quantitative theory of sleep, which explains the origins, features, mechanisms and functions in a detailed model^3^. One of the most studied, and yet debated^4^ functions of sleep is memory consolidation^5^ but evidence for this theory comes exclusively from human and laboratory rodent data, except for some results on arthropods^6^. Variation exists in the nature and the amount of sleep found in non-human species, and these variations suggest that functions of sleep may differ across species^2^, calling for the integration of human and laboratory rodent research into a wider set of results from different animal species^7^. In an effort to widen the framework to study both the general features and functions of vertebrate sleep^8^, here we investigate the relationship between sleep and memory in domestic dogs. Although extensive research has been carried out on dogs’ sleep EEG with ‘traditional’ invasive methods^9^,^10^,^11^,^12^, which mostly focused on neurological conditions such as epilepsy^13^,^14^ and narcolepsy^15^, this species has not been used previously to study the function of sleep in a way directly comparable to that of human studies. Dogs are one of the most interesting model species in comparative cognition research due their human-analogue social skills^16^,^17^ and their approximately 18–32 thousand years of domestication history^18^, during which they have adapted in evolutionary terms to the same environmental challenges as humans.

A non-invasive canine polysomnography method was developed for dogs^19^, and used here to investigate the differences in sleep EEG spectrum following a command learning (CL), and a non-learning (NL) task, respectively. Fifteen dogs participated in two polysomnography recordings (3-hour-long each), that immediately followed either CL, during which they had to associate unknown commands (unfamiliar words) to already known actions (sit and lie down), or NL, during which they were required to perform the same two actions after the usual (known) commands, in the very same way as in the CL task (see Experimental Procedures). After an initial adaptation session (where the polysomnography recording was not preceded by behavioural pre-treatment), dogs participated in both CL and NL conditions on two subsequent days in a counterbalanced order. Polysomnography recordings after the CL condition were followed by a post-sleep re-test session with the newly learned commands, in order to asses any change in the dogs’ performance, and its relation to sleep EEG spectrum. Importantly, this task allowed for the investigation of reward-related memory processing^20^, while current evidence for memory consolidation in non-human species mainly comes from aversive conditioning.

## Results and Discussion

### The effect of learning on sleep physiology

**Figure 1:**
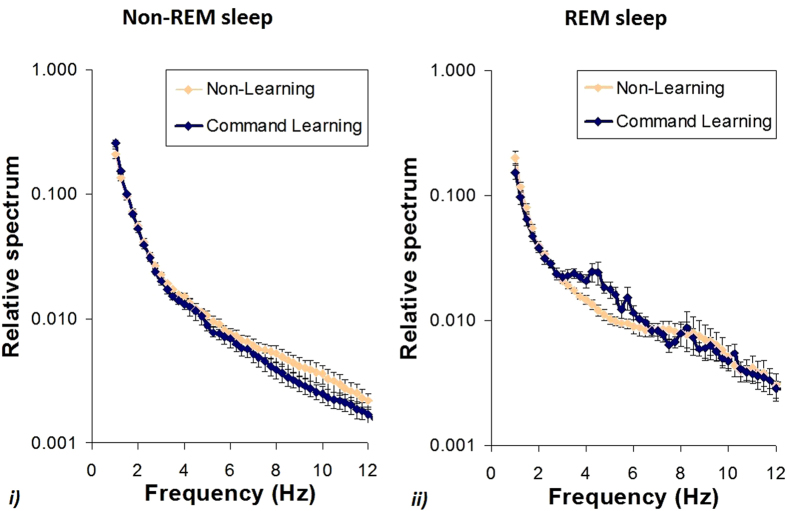
Relative power spectra (proportion of total power) for (**i**). Non-REM and (**ii**). REM sleep, following the command learning and the non-learning task. Bin-by-bin data (mean ± SE for the N = 15 participating dogs) are shown on a logarithmic scale for both Non-REM and REM sleep.

The relative EEG spectrum (proportion of total power) was first calculated for 4 Hz frequency ranges. This showed a redistribution of EEG power in a way that Non-REM sleep delta (1–4 Hz) activity increased (t_(14)_ = 2.943, p = 0.011), while alpha (8–12 Hz) activity decreased (t_(14)_ = 2.225, p = 0.043), after the learning task. The decrease in theta (4–8 Hz) activity was not significant (t_(14)_ = 1.926, p = 0.075), and no difference was found in beta (12–30 Hz) activity (t_(14)_ = 1.311, p = 0.211). The bin-by-bin (0.25 Hz resolution) analysis revealed that the relative delta activity increase occurred in the 1–1.5 and 2.75–3.25 Hz frequency ranges. There was a significant relative decrease in the 5–5.75 Hz (theta) range and in the 7–10.25 Hz (alpha) range (Fig. 1i). During REM sleep relative theta (4–8 Hz) activity increased after learning (t_(10)_ = 3.130, p = 0.011), while the relative decrease in delta (1–4 Hz) activity was not significant (t_(10)_ = 1.898, p = 0.087). No effect of learning on REM sleep EEG alpha (8–12 Hz; t_(10)_ = 0.539, p = 0.602), or beta (12–30 Hz; t_(10)_ = 1.305, p = 0.221) activity was found. According to the bin-by-bin analysis, there was a significant relative decrease in the 1.5–2 Hz (delta) frequency range after learning during REM sleep, while the relative increase in the 3.5–4 Hz (delta) frequency did not remain significant after correction for multiple comparisons. No significant bin-wise differences were found in the theta, alpha and beta ranges during REM sleep (Fig. 1ii). Spectral changes during Non-REM and REM sleep (when examining the difference between CL and NL conditions), were found to be related to each other in the theta range (pooled data, 4–8 Hz; r = −0.613, p = 0.045), but no such relationship was found for the other ranges (delta, alpha, beta; all p > 0.1). Within both sleep stages the change in slow activity (delta, 1–4 Hz), was negatively related to the change in fast activity (Non-REM alpha: r = −0.890, p < 0.001; beta: r = −0.730, p = 0.002; REM beta: r = −0.793, p = 0.004). Learning did not affect sleep macrostructure (see Supplemental Results), contrary to our expectations, but in line with some human studies, where similarly to our findings no differences were found between learning and non-learning conditions, regarding the time spent in different sleep stages^21^.

**Figure 2:**
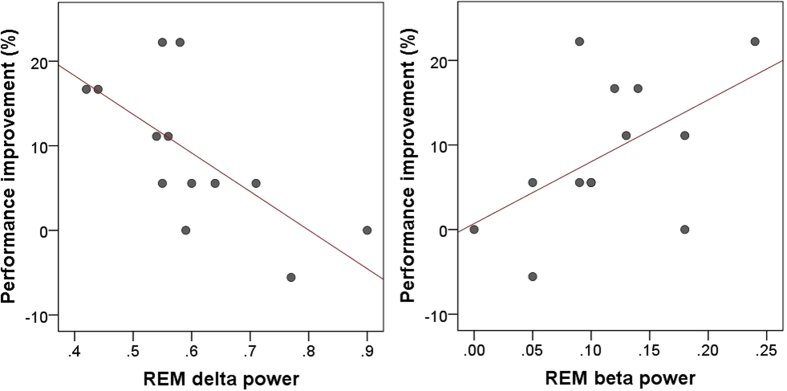
Relationship between performance improvement (the relative difference between pre-sleep and post-sleep performance) in the learning task, and relative delta power (left) as well as beta power (right) during post-learning REM sleep.

Behavioural data showed that subjects’ performance significantly increased after the 3-hour-long polysomnography recording compared to the pre-sleep baseline (t_(14)_ = 3.833, p = 0.002), although the performance increase was not related to sleep duration or any of the macrostructural variables (see Supplemental Results). However, evidence was found for a correlation between performance improvement and relative EEG spectrum power. Decreased REM sleep delta (1–4 Hz) activity (Pearson correlation; r = −0.683, p = 0.01), as well as increased REM sleep beta (12–30 Hz) activity (r = 0.536, p = 0.05), were related to higher performance (Fig. 2). There was no significant correlation of performance improvement with theta or alpha activity during REM sleep, or with any of the frequency ranges during Non-REM sleep.

These results provide the first evidence that learning new commands influences sleep EEG spectrum in dogs, and that the EEG spectrum during sleep is predictive of memory performance. Although “memory” is often used as a unitary term in the literature, it is not a single entity, and while in the case of humans there is a widely accepted distinction between declarative and non-declarative memory, we know little about how learning in non-human species fits into these categories. Our results suggest that command learning in dogs influences both REM and non-REM sleep, with the former being traditionally associated with non-declarative and the latter with declarative memory consolidation^22^. During non-REM sleep an increased delta power was found after learning, which is consistent with human data^23^,^24^.

Theta activity is typically thought to be implicated in many aspects of memory processing and consolidation, mostly due to the neuronal re-play of memories in the hippocampus during REM sleep^25^, but the direction of this relationship is controversial (e.g. in humans, learning of word pairs was reported to enhance theta activity during REM sleep^26^, however, mice exhibited reduced REM sleep theta activity after fear conditioning^27^). The present study also provided inconsistent results in the case of dogs, with some indications for increased theta activity during REM sleep after learning, and also reduced theta activity during non-REM sleep. However, these two changes were found to be functionally related, that is in line with the predictions of the two-stage model suggesting that subsequent occurrence of non-REM and REM sleep is essential for memory consolidation^28^. A decrease in alpha activity during non-REM sleep was also found, which together with the fact that alpha activity was negatively related to slow wave activity, might signal an increase in sleep depth after learning^29^.

### The effect of sleep and awake activity on learning

Having demonstrated learning-induced changes in sleep EEG spectrum and a relationship between sleep and memory formation in dogs, in the second experiment we aimed to test how post-learning activities (sleep or awake) influenced memory consolidation. A group of task-naïve adult pet dogs (n = 53) participated in the previously described command learning task (CL), during which their learning performance (Baseline) was assessed (see Experimental Procedures). After this, subjects were randomly assigned to four short (1 h) retention interval conditions (RIC) (n = 12–14/group). These either included sleeping, or one of three awake activities of varying physical and mental intensity: on-leash walk (physical activity with minimal cognitive interference), learning an unrelated task (low physical activity with high cognitive interference), playing with a dog toy Kong® while lying on the floor (minimal physical activity, high emotional arousal). Subjects’ performance in response to previously known commands was also assessed in order to control for obedience.

**Figure 3:**
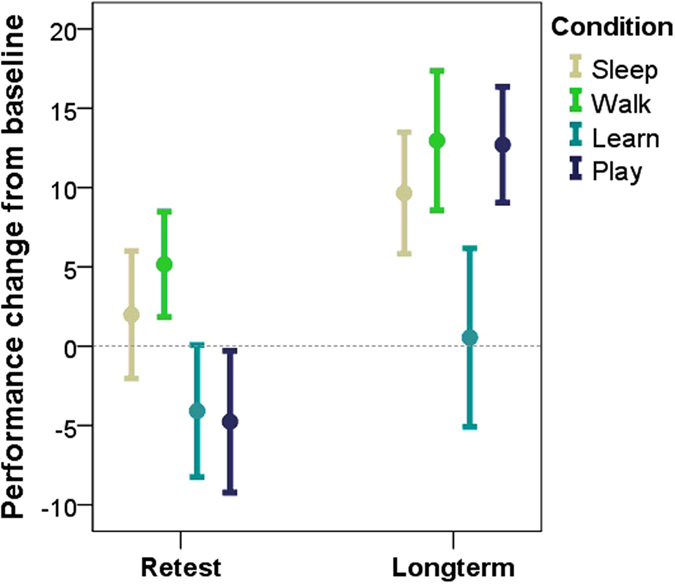
The differential learning patterns in the four retention interval conditions are revealed in subjects’ performance change (mean ± SE) at the Retest and Long-term occasions compared to Baseline. Values >0 indicate a performance improvement at the given occasion, while values <0 indicate a decreased performance.

Subjects in the four conditions did not differ in obedience (F_(3)_ = 0.799, p = 0.512), nor in baseline learning performance (F_(3)_ = 1.812, p = 0.157). Subjects were retested on the newly learned commands immediately after the retention interval (Retest), and after one week (Long-term), in order to assess short- and long-term memory effects of the different RICs. A Generalized Linear Mixed Model (Poisson Log; Table 1) showed that, as expected, performance was influenced by the interaction of test occasion (Baseline, Retest, Long-term) × RIC (χ^2^_(4)_ = 14.435, p = 0.006), suggesting that differential learning patterns emerged as a consequence of the different activities following the initial learning task (Fig. 3).

Subjects’ obedience also influenced their performance in interaction with the other two factors (Occasion × RIC × Obedience: χ^2^_(4)_ = 16.332, p = 0.003; RIC × Obedience: χ^2^_(2)_ = 9.037, p = 0.011; Fig. S1). The effect of RIC was also significant as a main effect (χ^2^_(2)_ = 8.020, p = 0.018), but the main effect of test occasion did not reach significance (χ^2^_(2)_ = 5.860, p = 0.053). The main effect of obedience (χ^2^_(1)_ = 0.770, p = 0.380) as well as its interaction with test occasion (Occasion × Obedience: χ^2^_(2)_ = 2.300, p = 0.317) were also non-significant. The pairwise post hoc analysis revealed that in the Sleep condition, despite a tendency towards performance improvement, there was no difference between the post-sleep retest and the baseline (p > 0.05). This result seemingly contradicts the findings of our polysomnography study (see Exp. 1 above), where dogs’ performance increased after 3 hours of sleep, but can probably be attributed to the difference in the length of the retention interval (3 hours vs. 1 hour), as longer sleep durations have been found to yield greater memory improvements in humans^30^. Future studies should determine the optimal amount of sleep needed to benefit memory and how this might generalize across species.

However, subjects in the Sleep condition did improve in the long run; they performed better when tested on the Long-term occasion compared to both Baseline (p < 0.001) and Retest (p < 0.001). This suggests that memory consolidation after learning occurred during the subjects’ usual night-sleep at home. This is in line with previous findings showing that in the absence of interfering learning experience, sleep does not need to occur immediately after learning for memory consolidation to take place^31^ but should happen on the same day as the initial training^32^. Subjects in the Walk condition showed the same learning pattern: there was no difference between Baseline and post-walk Retest (p > 0.05), but the Long-term performance was significantly higher (compared to both Baseline: p < 0.001; and Retest: p < 0.01). This suggests that being awake *per se* does not interfere with long-term memory formation in dogs. Similar claims have been made for humans^33^, suggesting that slow EEG oscillations during non-sleep resting state activity (mind-wandering) also facilitates memory consolidation.

Dogs that learned an unrelated task during the retention interval (Learning condition), not only remained at their baseline performance on the Retest occasion (p > 0.05), but also did not improve after a week (Baseline vs. Long-term: p > 0.05), suggesting that an interfering learning experience impedes memory consolidation for the previously learned information. In the Play condition subjects’ performance decreased at Retest compared to Baseline (p < 0.001), which is indicative of emotional arousal having a deteriorative effect. However, subjects in this condition also performed better on the Long-term occasion compared to both Baseline (p < 0.001) and Retest (p < 0.001), suggesting that these subjects also benefited from the at-home night sleep after learning, and that play did not interfere with memory consolidation, but impacted on other domains (e.g. attention), which are necessary for performance during re-test.

The results of these two studies provide the first evidence of the interrelated effect of sleep and learning in dogs, suggesting that a sleep-dependent memory consolidation takes place in this species. Further studies should determine if sleep and memory in dogs is similarly modulated by individual variation, as in the case of humans. For example if age-related changes in sleep-wake pattern^12^, EEG spectrum^19^ and memory function^34^ lead to memory consolidation differences in old dogs. Functional analogies in awake functioning between dogs and humans have already been proposed both at the behavioural^35^ and neural^36^ level. Our results open up the possibility that dogs’ human-analogue social learning skills might be related to sleep-dependent memory consolidation.

## Methods

### Ethic statement

Research was carried out in accordance with the Hungarian regulations on animal experimentation and the Guidelines for the use of animals in research described by the Association for the Study Animal Behaviour (ASAB). The Hungarian “Animal Experiments Scientific and Ethical Committee” issued a statement (under the number PE/EA/853–2/2016), approving our experimental protocol by categorizing it as a non-invasive study that causes less pain or suffering than the equivalent of inserting a needle. All owners volunteered to participate in the study.

### The effect of learning on sleep physiology

Subjects (N = 15 adult pet dogs, mean age ± SD: 3.67 ± 1.91; 8 males, 7 females; from 9 breeds and 3 mixed breeds), participated in 3-hour-long polysomnography recordings (according to the protocol described in ref. 19), for a total of three occasions (see Table S1). The first occasion was a 3-hour-long adaptation sleep, followed by a command learning (CL) and a non-learning (NL) occasion in a counterbalanced order (on three different days). In CL dogs were taught to perform two already known actions (sit and lie down), using unfamiliar commands (English phrases instead of the familiar Hungarian ones). The training procedure followed a standardized schedule and was concluded with an 18-trial baseline test session (for details see Supplemental Experimental Procedures). In the NL task dogs had to execute the same sequence of “Sit!” and “Lie down!” actions, but the experimenter always used the familiar commands (i.e. the Hungarian phrases for sitting and lying down), accompanied by the familiar hand signals (see Supplemental Experimental Procedures for details). Both the CL and NL tasks were followed by a 3-hour-long polysomnography recording. In the CL occasion, the polysomnography recording was followed by an 18-trial session where the dog had to execute the previously learned English commands (Retest).

Sleep recordings were visually scored according to standard criteria^19^ in 20 s epochs. Artefact rejection was carried out manually on 4 s epochs before further automatic analyses on all recordings. Average power spectral densities (1 Hz to 30 Hz) were calculated by a mixed-radix Fast Fourier Transformation (FFT) algorithm, applied to the 50% overlapping, Hanning-tapered 4 sec windows of the EEG signal of the Fz-Cz derivation. Relative power spectra were calculated separately for Non-REM and REM sleep for both the CL and NL occasions as proportion of total (1–30 Hz) power. The two conditions were compared with regard to the four frequency ranges of delta (1–4 Hz), theta (4–8 Hz), alpha (8–12 Hz) and beta (12–30 Hz), and additionally a bin-by-bin analysis was carried out on the full (1–30 Hz) spectrum with 0.25 Hz resolution.

Behavioural data was obtained from the learning task; the percent of correct actions was calculated for both the Baseline and the Retest sessions (18 trials each). The difference between the re-test and test sessions (improvement during sleep), was correlated with the relative spectrum in the four frequency ranges of delta (1–4 Hz), theta (4–8 Hz), alpha (8–12 Hz) and beta (12–30 Hz), for both Non-REM and REM sleep.

### The effect of sleep and awake activity on learning

Subjects (N = 53 adult pet dogs, mean age ± SD: 3.89 ± 2.59; 22 males, 31 females; from 21 breeds and 25 mixed breeds) participated in the command learning task (CL) described in Exp. 1. The CL was concluded with a 18-trial Baseline test session and followed by a 1-hour-long retention interval (RI) during which dogs participated in one of the following activities according to the condition they were quasi-randomly allocated: (1) sleeping in their owners’ parked car (N = 14); (2) walking around the university campus on leash (N = 14); (3) learning new commands with the owner in 10–minute-long sessions (N = 12); (4) playing with a Kong^®^ (N = 13). After the RI, dogs participated in an 18-trial Retest session as well as an 18-trial Obedience session with the known Hungarian commands. Approximately one week (mean ± SE: 7.64 ± 0.43 days) after the first occasion, dogs returned for another session of 18 trials to assess their long-term memory (Long-term; Table S2).

The percentage of correct actions was coded for the Baseline, Retest, Obedience and Long-term sessions respectively. A Generalized Linear Model (Poisson loglinear) was run with performance as the dependent variable, Occasion (Baseline, Retest, Longterm) and RI condition (Sleep, Walk, Learn, Play) as factors and Obedience as covariate.

## Additional Information

**How to cite this article**: Kis, A. *et al*. The interrelated effect of sleep and learning in dogs (*Canis familiaris*); an EEG and behavioural study. *Sci. Rep.* **7**, 41873; doi: 10.1038/srep41873 (2017).

**Publisher's note:** Springer Nature remains neutral with regard to jurisdictional claims in published maps and institutional affiliations.

## Supplementary Material

Supplementary Information

## Figures and Tables

**Table 1:** Mean ± SE performance (% of correct trials) of subjects in the different retention interval conditions (RICs).

*RIC*	Obedience	Test occasion		
		Baseline	Retest	Long-term
Sleep	83.73 ± 3.88	57.54 ± 3.33	59.52 ± 4.14	67.77 ± 3.52
Walk	85.32 ± 4.18	49.21 ± 4.03	54.37 ± 3.91	61.11 ± 3.53
Learn	78.24 ± 4.61	55.93 ± 2.60	51.85 ± 4.40	56.48 ± 4.58
Play	74.24 ± 5.31	48.99 ± 4.29	43.94 ± 8.46	63.13 ± 5.72

Obedience, Baseline, Retest and Long-term performances are given as the percentage of correct responses in each of the 18-trial sessions.
