# Supplementary material for: The interrelated effect of sleep and learning in dogs (Canis familiaris); an EEG and behavioural study
Source: Sci Rep. 2017 Feb 6;7:41873. doi: 10.1038/srep41873 (PMC5292958; doi:10.1038/srep41873)
Supplement: Supplementary Information [file srep41873-s1.doc]

**SUPPLEMENTAL INFORMATION**

**The interrelated effect of sleep and learning in dogs *(Canis familiaris)*; an EEG and behavioural study**

Anna Kis^1^*; Sára Szakadát^2^; Márta Gácsi^3,4^; Enikő Kovács^5^; Péter Simor^6^; Csenge Török^7^; Ferenc Gombos^8^; Róbert Bódizs^2,8^; József Topál^1^;

^1^ Institute of Cognitive Neuroscience and Psychology, Hungarian Academy of Sciences

^2^ Institute of Behavioural Sciences, Semmelweis University

^3^ MTA-ELTE Comparative Ethology Research Group

^4^ Department of Ethology, Eötvös Loránd University

^5^ Department of Ecology Faculty of Veterinary Sciences, Szent István University

^6^ Department of Cognitive Science, Budapest University of Technology and Economics

^7^ Institute for Health Promotion and Sport Sciences, Faculty of Education and Psychology, Eötvös University

^8^ Department of General Psychology, Pázmány Péter Catholic University

* vargane.kis.anna@ttk.mta.hu

**Supplemental Experimental Procedures**

EXPERIMENT 1 (The effect of learning on sleep physiology)

*Subjects*

Our subjects were 15 adult pet dogs (1−8-year-old; mean age ±SD: 3.67±1.91; 8 males, 7 females) from 9 breeds (Border collie, Golden retriever, Belgian shepherd, Briard, Labrador retriever, Malinois, Miniature Schnauzer, Poodle and Puli) and 3 mixed breeds. The body weight of the dogs ranged from 7.5 kg to 34 kg with a mean±SD of 21.03±8.55 kg. Owners were recruited on a voluntary basis and they were informed in detail about the aim, circumstances and features of the experiment in advance. In order to be eligible for the study, dogs had to be able to execute “sit” and “lie down” commands, and to be familiar with the hand signals for these commands. None of the dogs had any previous experience with the English commands that were used for the Command Learning task (see later), although we did not have such an exclusion criteria, dogs which were familiar with the English commands, would have been tested with other unfamiliar commands.

*Procedure*

Subjects participated in 3-hour-long polysomnography recordings on a total of three occasions (see **Table S1** for the summary of experimental steps). The first occasion was an adaptation sleep to avoid any effects of the unfamiliar environment of the testing situations − a phenomenon known as first-night effect in human literature^1^. Following the adaptation occasion (1−35 days afterwards), a command learning (CL) and a non-learning (NL) occasion followed in a counterbalanced order (8 dogs with CL first, 7 dogs with NL first). The recordings were scheduled for the afternoon (starting time varied across dogs from 12pm to 18pm), as apart from night time, dogs, similarly to humans, show the highest propensity to sleep during the afternoon^2^. The maximum difference between the starting time of the two recordings within one subject was 1 hour, and we controlled for the differences between weekdays vs. weekends as well (both the CL and NL occasion had to be either during the week or at the weekend). All dogs were awake during the period directly preceding the experiment, as being asleep before learning has an effect on memory consolidation^3^. Additionally, dogs followed their normal daily routine and were not sleep-deprived, as this also could have impacted on memory performance^4^. Pre-sleep brain activity during learning and recall can also affect memory consolidation^5^, however in our setup recording EEG during the learning task was not feasible.

The polysomnography recording followed our recently developed and validated canine protocol^6^. Surface attached scalp electrodes were placed over the anteroposterior midline of the skull (Fz, Cz, Pz) (electroencephalography, EEG), and on the zygomatic arch (*os zygomaticum*), next to the left eye (electrooculography, EOG). The ground electrode was placed on the left *musculus temporalis*. Fz and EOG derivations were referred to Cz. Electrodes were placed bilaterally on the *musculus iliocostalis dorsi* for electromyography (EMG), and over the second rib for electrocardiography (ECG). Respiratory movements were also monitored. Gold-coated Ag│AgCl electrodes fixed with EC2 Grass Electrode Cream (Grass Technologies, USA) were used for the recordings. Impedances for the EEG electrodes were kept below 15 kΩ. Signals were collected, pre-filtered, amplified and digitized at a sampling rate of 249 Hz/channel, by using the 30 channel Flat Style SLEEP La Mont Headbox, with implemented second order filters at 0.5 Hz (high pass) and 70 Hz (low pass), as well as the HBX32-SLP 32 channel preamplifier (La Mont Medical Inc., USA).

Immediately before the recording dogs participated in the CL or the NL task with the experimenter in the presence of their owner. In the CL session the dog had to learn to execute two known actions: “Sit!” and “Lie down!” on new commands (English commands instead of the familiar Hungarian ones). The teaching session always followed the same pattern and contained 6 blocks:

(1) executing both actions following the corresponding Hungarian commands without hand signals – 4 trials (in order to ensure that the dog knew the verbal commands)

(2) executing both actions following the corresponding Hungarian commands accompanied by the known hand signals – 4 trials

(3) executing the first action (“Lie down!”) following the English command AND the known hand signal – 10 trials (associative learning)

(4) executing the first action following the English command without hand signal – 10 trials

(5) executing the second action (“Sit!”) following the English command AND the known hand signal – 10 trials

(6) executing the second action following the English command without hand signal – 10 trials.

If the dog correctly performed the requested action within 5 seconds the experimenter praised it and gave it a treat. If the dog did not perform the correct action or offered another action, the experimenter moved away (so that the dog followed her and was again in a standing position), and repeated the same command until the dog executed it properly (and then the experimenter praised it and gave a treat).

The learning phase was followed by the Baseline test session, where the dog had to execute the previously learned English commands without hand signals; 18 trials of “Sit!” and “Lie down!” in a fixed pseudorandom order (LLSLSSLLSLSSLLSLSS).

In the NL situation dogs had to execute the same sequence of “Sit!” and “Lie down!” actions (6 blocks), but the experimenter used the familiar Hungarian commands accompanied by the familiar hand signals for blocks 3-6. This was followed by an 18-trial-long control session, during which the experimenter again used the familiar Hungarian commands accompanied by the familiar hand signals.

During the CL occasion, the polysomnography recording was followed by a re-test session, where the dog had to execute the previously learned English commands without hand signals; 18 trials in a fixed pseudorandom order. All the trials were video recorded.

| *1^st^ occasion* | *Counterbalanced order (2^nd^ / 3^rd^)* | |
| --- | --- | --- |
| **Adaptation** | **CL occasion** | **NL occasion** |
|  | Learning session  (6 blocks) | Known commands  (6 blocks) |
|  | Baseline session  (18 trials) | Control session  (18 trials) |
| Polysomnography recording (3 hours) | Polysomnography recording (3 hours) | Polysomnography recording (3 hours) |
|  | Re-test session  (18 trials) |  |

**Table S1.** Summary of the steps of the adaptation, CL and NL occasions that all the subjects participated in

*Data analysis*

Sleep recordings were visually scored (using a custom-made software developed by our laboratory: Fercio’s EEG Plus © Ferenc Gombos 2009-2016), according to standard criteria^6^ in 20 s epochs, by two experienced sleep researchers who inspected the EEG, EOG, ECG, EMG and Respiration channels. This coding resulted in high inter-rater reliability (Cohen’s κ=0.91 based on double scoring of 9 recordings with 10 randomly chosen epochs / recording), for the wakefulness stage, the drowsiness stage, the Non-REM stage and the REM sleep stage. Different stages of Non-REM sleep (e.g. Stage 2 and Slow Wave Sleep) were not coded separately as sleep spindles are not as salient in dogs’ EEG recordings as in humans, thus a satisfactory inter-rater reliability could not be achieved for this distinction^6^. Raters were always blind to subject and condition details.

The following macrostructural data was exported from the resulting hypnograms: Sleep Duration (min), Sleep Efficiency (%), Waking After Sleep Onset (after first drowsiness, min), Sleep Latency (until first drowsiness, min), Drowsiness Duration (min), Non-REM duration (min), REM duration (min), REM Latency (from first drowsiness, min), and average Sleep Cycle Duration (min). In addition, Rapid Eye Movement Density during REM sleep was manually scored, by coding every 2-second-long interval as containing a rapid eye movement or not (in the case of the n=11 dogs that had REM sleep on both occasions). Sleep recordings following the learning versus the control task were compared with regard to all these variables (paired samples t-test).

Artefact rejection was carried out manually on 4 s epochs before further automatic analyses on all recordings. Average power spectral densities (1 Hz to 30 Hz) were calculated by a mixed-radix Fast Fourier Transformation (FFT) algorithm, applied to the 50% overlapping, Hanning-tapered 4 sec windows of the EEG signal of the Fz-Cz derivation. The Fz-Cz derivation was used due to practical reasons, as the head anatomy of dogs did not allow for other derivations to be used ^6^. In humans, the reported changes in EEG spectrum after a learning task, are often localized to specific brain regions that our canine design did not allow us to investigate. However, a change in the Fz derivation could be expected after the command learning task, as in humans the frontal region has been implicated in memory consolidation after a word learning task^7^. Power spectra were calculated separately for NREM and REM sleep on 0.25 Hz frequency bins for both the learning and non-learning occasions. No spectral analysis was carried out for the EEG signal of the wake and drowsiness stages, due to the high proportion of artefacts (resulting from muscle tone). Relative EEG power was calculated for each frequency bin as the percent of total power (e.g. the absolute power value of the given bin divided by the sum of absolute power values for the 1-30 Hz frequency range).

The CL and NL occasions were compared with regard to the relative spectrum power in the four frequency ranges of delta (1-4 Hz), theta (4-8 Hz), alpha (8-12 Hz) and beta (12-30 Hz). Additionally, a bin-by-bin analysis was carried out on the full (1-30 Hz) spectrum with 0.25 Hz bins. In order to address the issue of multiple comparisons, a Rüger correction was used on the bin-by-bin results^8^. Rüger’s areas are defined as sets of conventionally significant (p<0.05) results, which are accepted or rejected as significant as a whole, instead of individual results of statistical tests. Taking the results of the statistical tests as a matrix, we defined Rüger’s areas along the dimension of frequency bins. Starting from the lower frequencies, a Rüger’s area was the range of all the neighbouring, consecutive frequency bins, which contain a significant result surrounded by bins containing non-significant results. After defining these areas of significance, the number of significant results within the area was calculated, and we investigated whether at least half of these results were significant at least at half of the conventional p=0.05 significance level (that is, whether they were below 0.025), and at least one third of them were significant at least at a third of the conventional p=0.05 significance level (below 0.0167). If both of these conditions were fulfilled, the area as a whole was considered significant.

A correlation analysis was carried out in order to see if the differences in relative spectrum between CL and NL occasions were related in NREM and REM sleep in the four frequency ranges of delta (1-4 Hz), theta (4-8 Hz), alpha (8-12 Hz) and beta (12-30 Hz) respectively (Pearson correlations). Furthermore, within-sleep-stage (NREM and REM) correlations were investigated (on CL − NL differences) between the different frequency ranges (delta, theta, alpha, beta), in order to test the relatedness of fast and slow-wave activity change (Pearson correlations).

Data from the absolute spectra could not be used for “conventional” comparisons as the absolute EEG power differed across subjects in several magnitudes. This high individual variation can most probably be attributed to the extreme within-species heterogeneity that characterizes dogs, and specifically it might be due to the large individual differences in skull morphology (e.g. the thickness of the parietal and frontal bone). Future research should confirm the effect of such individual variation on EEG signal. Thus, the proportion of absolute spectral power of CL/NL occasions was calculated for each subject (separately for NREM and REM sleep), and compared to 1 (no change), by using one-sample t-tests in a 0.25 bin-by-bin resolution with a Rüger correction. Subjects were only included in the spectral analysis if they exhibited more than 10 minutes of sleep in the given stage (NREM / REM), consequently the final sample consisted of N=11 dogs for NREM and N=10 dogs for REM.

Behavioural data was obtained from the CL task, and the percent of correct actions was calculated for the last block of 18 trials (English commands without hand signals in a pseudorandom order), for both the pre-sleep (Baseline), and the post-sleep (Retest) sessions. A correct action was coded if the dog executed the “Sit!” and “Lie down!” action associated with the given command within 5 seconds; if the dog performed the correct action after the given command when it was repeated, that trial was coded as incorrect (the repetition was only necessary to make sure that subjects in the CL and NL conditions received the same amount of food reward). The difference between the Retest and Baseline sessions (improvement during sleep), was correlated with the macrostructural variables, and with the relative spectrum in the four frequency ranges of delta (1-4 Hz), theta (4-8 Hz), alpha (8-12 Hz) and beta (12-30 Hz), for both NREM and REM sleep.

All statistical procedures were carried out with SPSS22.

EXPERIMENT 2 (The effect of sleep and awake activity on learning)

*Subjects*

Our subjects were 54 adult (1−10-year-old) pet dogs (22 males, 32 females), from 21 breeds (Argentine Pila dog, Belgian shepherd, Bernese Mountain dog, Bichon Bolognese, Bichon Havanese, Border collie, Chinese Crested dog, Dachshund, Fox terrier, German shepherd, Golden retriever, Hungarian greyhound, Hungarian Vizsla, Labradoodle, Labrador retriever, Miniature Schnauzer, Papillion, Pumi, Schipperke, Shiba Inu, and Whippet) and 25 mixed breeds. Owners were recruited on a voluntary basis and they were informed in detail about the experiment in advance, however they were blind to the objectives and hypothesis of the study. In order to be eligible for the study, dogs had to be able to execute “sit” and “lie down” actions on command and be familiar with the hand signals for these commands (reported by the owners). One subject (a 1-year-old female Hungarian Vizsla), was excluded as it failed the 50% criteria in the obedience task, that consisted of executing the “sit” and “lie down” commands. None of the dogs had any previous experience with the English commands that were used for the Command Learning task (see later).

*Procedure*

Subjects participated in the command learning (CL) task described in Study 1 (see **Table S2** for the summary of experimental steps), where they had to perform two known actions: “Sit!” and “Lie down!” on hearing new commands from the experimenter (English commands were used instead of the familiar Hungarian ones). The teaching session always followed the same pattern (in 6 blocks), and was concluded with a baseline test session of 18 trials (consisting of English commands without hand signals), in a fixed pseudorandom order. The teaching session was followed by a 1-hour-long retention interval, during which dogs participated in one of the following activities according to the retention interval condition (RIC) they were quasi-randomly allocated.

(1) Sleep (N=14): immediately after the CL session, subjects were returned to their owners’ car or to an empty room at the University, where they quietly rested quietly for one hour at their usual place (e.g. in their dog cage with their blanket), in the presence of their owner. All owners reported that their dogs were sleeping during the retention interval, that is plausible based on the macrostructural data of experiment 1 where dogs fell asleep with a mean±SE sleep latency of 10.91±2.18 minutes (maximum: 36.33 minutes).

(2) Walking (N=14): immediately after the teaching session, subjects were taken by their owners for a one-hour-long on-leash walk around the university campus. While our goal with this RIC was to induce the least possible cognitive interference while keeping the dogs awake, it cannot be excluded that as dogs explored their environment during the walk some interference did occur. In order to minimize this (without causing stress to the animals), dogs were walked on a leash and thus not allowed to explore freely, nor to interact with other dogs or humans.

(3) Learning (N=12): during a one-hour interval dogs participated in three 10-minute-long sessions where they were taught previously unknown actions by their owner, with luring as a training method. The first such session commenced 5 minutes after the beginning of the retention interval, and two 10-minute-long breaks were inserted in between the three sessions, during which the dog was quietly resting. The retention interval was concluded with another 5 minutes of quiet resting. The experimenter was passively present during the whole one-hour interval.

(4) Play (N=13): subjects received a dog toy (Kong®) filled with food, for a total of three times during the one-hour-long interval. The first 5 minutes of the interval were spent with quiet resting, then the dog received the Kong® filled with food and was allowed to interact with it. It took the dogs approximately 10 minutes to get the food from the Kong®, then for another 10 minutes they were quietly lying and/or chewing on the empty Kong® before the experimenter re-filled it with food.

The length of the retention interval (1 hour) was chosen to be the maximum that dogs could spend with learning and resting without falling asleep, which did not cause stress to them. Dog were randomly allocated to one of the conditions in which they fulfilled the necessary criteria. In the Sleep condition, owners had to be willing to transport the dog in their car to the University, and/or bring the dogs’ usual sleeping place (e.g. a blanket). In the Walking condition, owners had to be willing to walk their dogs for one hour. In the Learning condition, owners had to be confident in teaching new commands to their dog with luring as a training method. In the Play condition, dogs had to be familiar with the dog toy Kong®. Owners were initially asked about these criteria and were then randomly allocated to one of the possible conditions. Dogs assigned to the four retention interval conditions (RIC) did not differ in age (F_(3)_=0.337, p=0.798).

We did not include a ‘Resting’ awake condition that would have been the closest match to the Sleep condition (which is generally used in most human memory consolidation studies), as preventing dogs from falling asleep while requested to stay in a laying position for one hour would presumably induce stress in the animals. Stress is known to have an impact on memory^9^, and also raises animal welfare issues, thus we decided to avoid such a condition. These concerns are supported by a study^10^ showing that in a discrimination learning task using arbitrary stimuli (blue basket with white dots filled with woodchip vs. green box with black stripes filled with cat litter) dogs took longer to re-learn the discrimination one day after the initial training, if the latter was followed by 30 minutes of resting (during which dogs were required to lie down on a dog bed and were called by their name, and/or touched to prevent them from falling asleep), compared to a group that engaged in 30 minutes of physical activity including walking and playing (fetching a ball, running after Frisbees, and playing tug-of-war, depending on each dog’s preferred playing style).

After the retention interval, dogs participated in a Retest session, where 18 trials (consisting of English commands of “Sit” and “Lie down” without hand signals), were initiated in a fixed pseudorandom order (LLSLSSLLSLSSLLSLSS). An additional obedience session was then administered, that consisted of the same 18 trials again in a fixed pseudorandom order, but with the known Hungarian commands accompanied by the known hand signs, in order to assess the obedience of the dogs.

After the first occasion, dogs returned around a week later for another (Long-term) session of 18 trials (English commands without hand signals), in the same fixed pseudorandom order, in order to test their long-term memory. The minimum time elapsed between the two session was 4 days, with a mean±SE of 7.64±0.43 days. Three subjects (1 from the Sleeping and 2 from the Learning condition) did not return for the long-term session, thus in these cases only data from the first occasion was included in the analysis. Owners did not receive any specific instructions to follow during the time between the two study occasions, but they were asked to follow their normal routine. Owners were not explicitly forbidden to use the newly learned words, to avoid them feeling as if the second occasion was a test, for which they needed to practice. After the experiment all owners reported not having practiced the new commands in-between the occasions.

| *1st occasion* | *2nd occasion* |
| --- | --- |
| Learning session  (6 blocks) |  |
| Baseline test session  (18 trials) |  |
| Retention interval (1 hour) Sleep / Walk / Learn / Play |  |
| Retest session  (18 trials) | Long-term session  (18 trials) |
| Obedience session  (18 trials) |  |

**Table S2.** Summary of the steps of each occasion subjects participated in

*Data analysis*

The percent of correct actions (if the dog executed the “Sit!” and “Lie down!” action associated with the given command within 5 seconds), was coded from the video recordings (which were blind to the subject condition) for the Baseline, Retest, Obedience and Long-term sessions respectively. A Generalized Linear Model (Poisson loglinear) was run with performance as the dependent variable, Occasion (Test, Retest, Long-term) and Condition (Sleep, Walk, Learn, Kong) as factors and Obedience as covariate.

**Supplemental Results**

In Experiment 1, the macrostructure of sleep after the command learning (CL) versus the non-learning (NL) task was compared, but no difference was found in any of the variables investigated: Sleep Duration (t_(14)_=1.258, p=0.229), Sleep Efficiency (t_(14)_=1.241, p=0.235), Waking After Sleep Onset (t_(14)_=0.983, p=0.342), Sleep Latency (t_(14)_=0.902, p=0.382), Drowsiness Duration (t_(14)_=0.189, p=0.853), Non-REM Duration (t_(14)_=1.162, p=0.265), REM Duration (t_(14)_=0.117, p=0.908), REM Latency (t_(14)_=0.938, p=0.363), Rapid Eye Movement Density (t_(11)_=0.703, p=0.497), and Sleep Cycle Duration (t_(14)_=0.200, p=0.844).

No relationship was found between performance improvement (difference between pre-sleep and post-sleep performance), and any of the sleep macrostructure variables: Sleep Duration (r=0.098, p=0.727), Sleep Efficiency (r=0.098, p=0.727),Waking After Sleep Onset (r=-0.121, p=0.667), Sleep Latency (r=-0.085, p=0.762), Drowsiness Duration (r=0.103, p=0.715), Non-REM Duration (r=0.166, p=0.554), REM Duration (r=-0.150, p=0.593), REM Latency (r=0.306, p=0.267), Rapid Eye Movement Density (r=0.037, p=0.910), and Sleep Cycle Duration (r=-0.409, p=0.130).

The analysis of the absolute spectrum showed that the proportion of EEG power during NREM sleep after CL/NL was above 1 (increased in CL) in the ranges of 1- 1.5 Hz (delta), and below 1 (decreased in CL) in the ranges of 5-5.5 Hz (theta), 6.5-13 Hz (alpha), and 17.75-18.25 Hz (beta). No significant change could be detected in REM absolute power (all p>0.07).

**
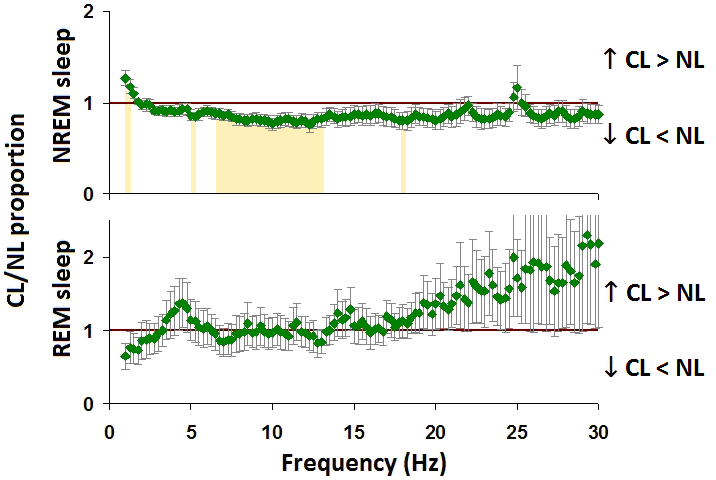
**

**Figure S1.** Proportion of sleep EEG absolute power after Command Learning / Non-Learning during NREM and REM sleep (mean±SE). Values >1 indicate an increase in absolute spectrum after Command Learning, while values <1 indicate a decrease in absolute spectrum after Command Learning. Frequency ranges that significantly differ from 0 are highlighted with yellow.

**Supplemental Figure**


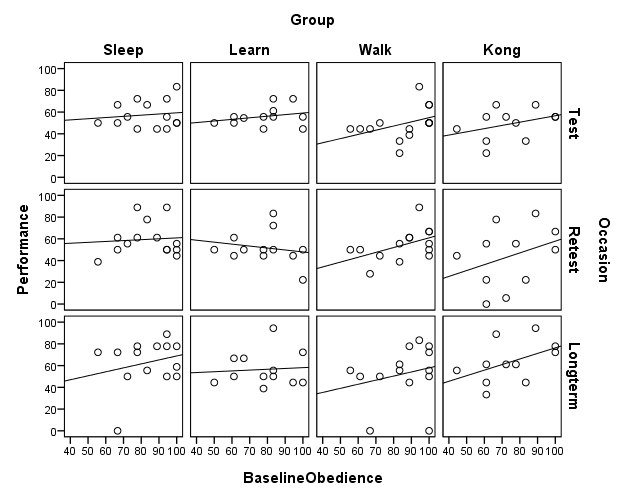


**Figure S2.** Relationship between subjects’ obedience and performance on the three test occasions in the different retention conditions.

**Supplemental References**

1. Agnew, H. W., Webb, W. B. & Williams, R. L. The first night effect: an EEG study of sleep. *Psychophysiology* **2,** 263–266 (1966).

2. Takahashi, Y., Ebihara, S., Nakamura, Y., Nishi, C. & Takahashi, K. Circadian sleep and waking patterns in the laboratory dog. *Sleep Res.* **1,** 144 (1972).

3. Grosvenor, A. & Lack, L. C. The effect of sleep before or after learning on memory. *Sleep* **7,** 155–167 (1984).

4. Yoo, S.-S., Hu, P. T., Gujar, N., Jolesz, F. a & Walker, M. P. A deficit in the ability to form new human memories without sleep. *Nat. Neurosci.* **10,** 385–92 (2007).

5. Heib, D. P. J. *et al.* Oscillatory theta activity during memory formation and its impact on overnight consolidation: A missing link? *J. Cogn. Neurosci.* **27,** 1648–1658 (2015).

6. Kis, A. *et al.* Development of a non-invasive polysomnography technique for dogs (Canis familiaris). *Physiol. Behav.* **130,** 149–156 (2014).

7. Schmidt, C. Encoding Difficulty Promotes Postlearning Changes in Sleep Spindle Activity during Napping. *J. Neurosci.* **26,** 8976–8982 (2006).

8. Abt, K. Descriptive data analysis: a concept between confirmatory and exploratory data analysis. *Methods Inf. Med.* **26,** 77–88. (1987).

9. Roozendaal, B., McEwen, B. S. & Chattarji, S. Stress, memory and the amygdala. *Nat. Rev. Neurosci.* **10,** 423–433 (2009).

10. Affenzeller, N., Palme, R. & Zulch, H. Playful activity post-learning improves training performance in Labrador Retriever dogs (Canis lupus familiaris). *Physiol. Behav.* **168,** 62–73 (2016).
